# Mortality risk in adults according to categories of impaired glucose metabolism after 18 years of follow-up in the North of Spain: The Asturias Study

**DOI:** 10.1371/journal.pone.0211070

**Published:** 2019-01-31

**Authors:** Jessica Ares, Sergio Valdés, Patricia Botas, Cecilia Sánchez-Ragnarsson, Sandra Rodríguez-Rodero, Paula Morales-Sánchez, Edelmiro Menéndez-Torre, Elías Delgado

**Affiliations:** 1 Asturias Central University Hospital, Endocrinology and Nutrition Department, Oviedo, Asturias, Spain; 2 Málaga Regional University Hospital, Endocrinology and Nutrition Department, Málaga, Andalucía, Spain; 3 San Agustín University Hospital, Endocrinology Department, Avilés, Asturias, Spain; Temple University, Lewis Katz School of Medicine, UNITED STATES

## Abstract

People who develop type 2 diabetes (T2D) are known to have a higher mortality risk. We estimated all-cause, cardiovascular, and cancer mortality-risks in our patient cohort according to categories of impaired glucose metabolism. This 18-year retrospective analysis included a region-wide, representative sample of a population aged 30–75 years. Age- and sex-stratified hazard ratios (HRs) were calculated for 48 participants with diagnosed T2D, 83 with undiagnosed T2D (HbA1c ≥6.5%, fasting glycemia ≥126 mg/dL, or glycemia after 75 g glucose load ≥200 mg/dL); 296 with prediabetes (HbA1c 5.7%-6.4%, fasting glycemia 100–125 mg/dL, or glycemia after 75 g glucose load 140–199 mg/dL), and 607 with normoglycemia. Over 18,612 person-years, 32 individuals with undiagnosed T2D, 30 with diagnosed T2D, 62 with prediabetes, and 80 with normoglycemia died. Total sample crude mortality rate (MR) was 10.96 deaths per 1,000 person-years of follow-up. MR of the diagnosed T2D group was more than 3-times higher and that of newly diagnosed T2D was 2-times higher (34.72 and 21.42, respectively) than total sample MR. Adjusted HR for all-cause mortality was 2.02 (95% confidence interval 1.29–3.16) and 1.57 (95% CI 1.00–2.28) in the diagnosed T2D group and the newly diagnosed T2D group, respectively. Adjusted HR for cardiovascular mortality in the T2D group was 2.79 (95% CI 1.35–5.75); this risk was greatly increased in women with T2D: 6.72 (95% CI 2.50–18.07). In Asturias, age- and sex-standardized all-cause mortality is more than 2-times higher for adults with T2D than for adults without T2D. The HR for cardiovascular mortality is considerably higher in T2D women than in normoglycemic women.

## Introduction

Worldwide mortality caused by diabetes reached four million in 2017 [1]. About half of diabetes-related mortality (48%) occurs in people younger than 60 years. Although a decrease in mortality caused by diabetes has been observed in Europe and North America [2], diabetes continues to reduce life expectancy by 6–8 years in people diagnosed at the age of 50 years. The majority (>50%) of diabetes-caused mortality is from cardiovascular diseases.

The association between diabetes and premature death has been confirmed by a review of 97 prospective trials with 820,900 people [3]. As a high percentage of type 2 diabetes (T2D) has not been diagnosed, the mortality risk in this population has not been sufficiently investigated. The DECODE study (13 European cohorts) defined the proportion of unknown diabetes to be between 39% and 70%, depending on sex and age [4]. According to the latest International Diabetes Federation Diabetes Atlas [1], it has been estimated that, globally, as many as 212.4 million people or half (50.0%) of all people aged 20–79 years with T2D are unaware of their disease.

T2D increases both cardiovascular and all-cause mortality [5]. Both appear to have a directly proportional relationship with glycemia [6]. Despite this, there is no homogeneity in different studies regarding the magnitude of the potential risks.

## Materials and methods

### The asturias study

The Asturias Study is a population-based prospective cohort study of T2D and cardiovascular risk factors whose framework is the population of Asturias (a region in the north of Spain). The first phase was conducted in 1998–1999, with the aim to determine the prevalence of T2D (both diagnosed and undiagnosed) in the Asturias population. Asturias is home to approximately 1 million people, mostly white. More than half of the population lives in urban areas. A two-stage, probability cluster sampling procedure was applied. First, we selected 15 of 75 Asturias districts for analysis. Second, we selected age- and sex-stratified random samples of adults aged 30–75 years from each district [7]. Participants were randomly selected from the general population.

The resulting sample included 1,875 people; 87 were excluded due to pre-specified criteria (e.g., type 1 diabetes mellitus, pregnancy, severe disease, hospitalization, and treatment with hyperglycemic drugs) and 162 were excluded due to lack of contact data. Our final sample included 1,626 people but only 1,034 (63.6% response rate) participated.

Healthcare Principality of Asturias Ethics Committee approved the study, and all participants gave written informed consent. A health survey was conducted within the sample to collect relevant information, including demographic data, smoking habits, physical activity, socioeconomic status, and family history of T2D. Cardiovascular risk factors were assessed using standardized methods based on World Health Organization (WHO) recommendations [8]. Height and weight were measured with the participant wearing light clothing and without shoes, and body mass index (BMI) was calculated (weight in kilograms divided by the square of the height in meters). Blood pressure (BP) was measured using a digital sphygmomanometer (OMROM MX3, OMROM Healthcare, Tokyo, Japan) with the person seated and at rest. The mean of two BP measurements, taken 1–2 min apart, was used for this analysis.

### Measurements

As we used data collected in 1998, the methodology is similar to that described by Valdés et al. in 2009 [9]. Oral glucose tolerance tests (OGTTs) were performed in all individuals without previously known T2D. Fifteen minutes after each blood extraction, the blood was centrifuged *in situ* using a portable centrifuge. The samples were transported daily in a portable refrigerator (4–6°C) for processing at the Clinical Biochemistry laboratory of the Hospital Universitario Central de Asturias. Glucose levels were measured after fasting and after the OGTT (glucose-hexokinase enzyme method, Hitachi 747 analyzer, Roche Diagnostics, Mannheim, Germany). Measurements were also made of concentrations of total cholesterol, high-density lipoprotein cholesterol, triglycerides (colorimetric enzyme method, Hitachi 747 analyzer, Roche Diagnostics, Mannheim, Germany), low-density lipoprotein cholesterol (LDL-C) (Friedewald equation), and glycohemoglobin (HbA1c) (high-performance liquid chromatography, Jokoh HS-10 automated HbA1c analyzer, computer interface RS232). Initial Japanese HbA1c measurements were standardized to International Federation of Clinical Chemistry (IFCC) values (Japanese Society of Clinical Chemistry HbA1c = 0.927 (IFCC HbA1c) + 1.73).

Diagnosed diabetes was defined according to self-reported medical diagnosis or the use of antidiabetic drugs. We used ADA (American Association of Diabetes) 2018 criteria to define categories of glucose metabolism: newly diagnosed diabetes (≥126 mg/dL/≥7.0 mmol/L fasting, ≥200 mg/dL/≥11.1 mmol/L 2-h post-glucose load, or HbA1c ≥6.5%); impaired glucose tolerance (IGT) (2-h post-glucose load during 75-g OGTT 140 mg/dL [7.8 mmol/L] to 199 mg/dL [11.0 mmol/L]); impaired fasting glucose (IFG) (fasting plasma glucose 100 mg/dL [5.6 mmol/L] to 125 mg/dL [6.9 mmol/L]); and NGT. Prediabetes includes IFG and/or IGT and/or HbA1c 5.7%-6.4% (39–47 mmol/mol).

Previous reports about the Asturias Study [7,9,10] used WHO 1999 criteria to define categories of glucose metabolism; the difference from the criteria used in this study is that IFG is between 6.1 and 7.0 mmol/L, and HbA1c is not considered a diagnostic parameter. Body fat percentage (BF%) was calculated by applying the Clinica Universidad de Navarra—Body Adiposity Estimator (CUN-BAE) predictive equation (11)(11)(11)(11)(11)(11)(11)[11]. Obesity was taken to be a BF%≥25% in men and≥35% in women.

### Follow up and mortality

In December 2016, the vital status of the initial cohort (studied in 1998–1999) was updated. Deaths were recorded by healthcare card data of Principality of Asturias. Cause of death for the 18-year follow-up was ascertained by our register of mortality. All deaths were coded according to the International Classification of Diseases, 10th Revision (WHO) [12]. Codes I00-I99 (cardiovascular diseases) or R96 (sudden death, unknown cause) defined cardiovascular mortality because, generally, sudden death is due to cardiovascular disease [13]. Codes C00-D48 defined cancer death.

### Statistical analysis

All calculations were performed with SPSS version 21.0 (SPPS Inc, 2013). The reported P-values were based on a two-tailed test with a limit of statistical significance set to P<0.05. Evaluation of the risk of death in the different categories of dysglycemia was done by classifying the people included in the registry of deaths into four groups according to the results of the OGTT during the first study (ADA 2018 criteria). Comparisons between groups for quantitative variables were done with an analysis of variance. The person-years of follow-up were estimated for each group, as was the number of events (deaths), by calculating the mortality rates for every 1000 inhabitant-years (95% confidence interval [CI]).

Cox regression analysis was used to analyze the accumulated impact curves and the corresponding hazard ratios (HRs) of death, adjusted for age, sex, and multivariable (adjusted for age, sex, BMI, history of high BP, smoking habits, LDL-C, glomerular filtration rate, and the prior presence of cardiovascular disease) in the different groups. Hazard ratios represent the ratio of death probabilities. For example, a hazard ratio of 2 for the diagnosed diabetes group means that this group has twice the risk of dying than the normoglycemia group.

## Results

### Baseline characteristics

The Asturias Study cohort included 1,034 individuals (58.7% with normoglycemia, 28.1% with prediabetes, 4.6% with known T2D, and 9.6% with newly diagnosed T2D). Individuals with previously known T2D were significantly older, less likely to smoke, had higher systolic BP, more previous history of cardiovascular disease, higher triglycerides, and lower glomerular filtration rate than individuals in the other groups. However, they had similar or even lower figures for total cholesterol and LDL-C than the general population without dysglycemia; these values were highest in the groups with prediabetes and undiagnosed DM. Likewise, there was a progressive increase in LDL-C across the groups of fasting hyperglycemia and HbA1c. Of note was the fact that the mean levels of HbA1c in the group with undiagnosed T2D were almost within the range of normality. Newly diagnosed T2D had the highest BF% (Table 1).

**Table 1:** Metabolic and cardiovascular risk parameters according to the different clinical categories of dysglycemia during the first phase of the study (1998–1999).

	Total population	Normoglycemia	Prediabetes	Unknown diabetes	Diagnosed diabetes	P-value
**Patients (n)**	1034	607	296	83	48	<0.001
**Men (%)**	473 (45.7)	238 (39.2)	160 (54.1)	47 (57.6)	28 (58.3)	<0.001
**Age (years)**	52.1±13.4	49.4±3	56.5±12	61.5±11.2	65.6±8.6	<0.001
**Smokers (%)**	293 (28.3)	177 (29.2)	65 (22)	19 (22.9)	10 (20.8)	<0.001
**Previous CVD (%)**	49 (4.7)	12 (2)	19 (6.4)	9 (10.8)	9 (18.8)	0.001
**Estimated body fat (%)**	34.6±8.1	33.6±7.7	35.6±8.4	37.7±7.7	36.0±9.7	<0.001
**BMI (kg/m**^**2**^**)**	27.6±4.7	26.5±4.3	28.9±4.4	30.5±4.8	28.9±5.7	<0.001
**SBP (mmHg)**	134.2±22.1	127.4±19.4	139.9±21	151.6±21.1	155.3±22.8	<0.001
**DBP (mmHg)**	83.7±13.5	80.1±12.4	87.5±13.7	91±13.2	91.8±11.6	<0.001
**Total cholesterol (mg/dL)**	223.45.5	223.9±40.6	236.4±43.4	240.7±38.4	235.9±95.4	<0.001
**HDL cholesterol (mg/dL)**	56.3±14.2	58.3±14.7	54.2±12.6	51.8±14.2	51.7±12.9	<0.001
**LDL cholesterol (mg/dL)**	149.6±37.0	145.7±36.6	156.3±38.4	158.6±33.5	141.3±30.9	<0.001
**TG (mg/dL)**	125.0±61.3	102.8±66.1	130.5±65.7	154.3±78.3	152.5±77.8	<0.001
**eGFR (ml/min)**	78.6±19.3	83.1±18.9	73.6±18.1	71.5±17	65.6±19.1	<0.001
**Fasting glucose (mg/dL)**	100.2±23.5	89.6±6.2	104.6±9.0	123.4±23.3	168±56.2	<0.001
**HbA1c (%)**	5.3±0.8	5±0.4	5.3±0.4	6±1	7.5±1.7	<0.001

Abbreviations: CVD, cardiovascular disease; BMI, body mass index; SBP, systolic blood pressure; DBP, diastolic blood pressure; HDL, high-density lipoprotein; LDL, low-density lipoprotein; TG, triglycerides; eGFR, estimated glomerular filtration rate; HbA1c, glycohemoglobin.

### Mortality by glucose tolerance categories

**Fig 1:**
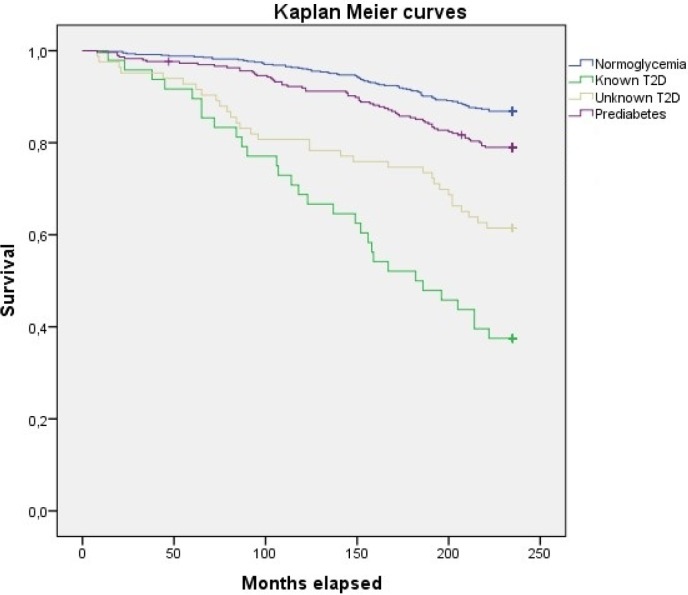
Accumulated all-cause mortality Kaplan-Meier curves for the groups with normoglycemia, prediabetes, unknown T2D and known T2D. Not adjusted model.

Over the 18 years of follow-up, 204 (19.8%) deaths were recorded. Deaths were classified in three groups according to etiology: cardiovascular, 74 (36.3%); cancer, 72 (35.3%); and other causes, 58 (28.4%). The crude rate for all-cause mortality was 10.96% (95% CI 9.55–12.57) per 1,000 person-years. Crude mortality rates for people without carbohydrate metabolism disorders were the lowest compared to those of people with prediabetes, known T2D, and unknown T2D (Table 2 and Fig 1).

**Table 2:** Crude and standardized hazard ratios and rates for all-cause mortality.

	Normoglycemia	Prediabetes	T2D			Total
			Unknown T2D	Known T2D	Total T2D	
**Patients (n)**	607	296	83	48	131	1034
**Deaths (n)**	80	62	32	30	62	204
**Deaths/1,000 person-years (95% CI)**	7.32 (5.72–8.92)	11.64 (8.74–14.54)	21.42 (14.00–28.84)	34.72 (22.3–47.13)	27.12 (21.14–34.79)	10,96 (9.55–12.57)
**HR (95% CI)**	1	1.68 (1.20–2.33)	3.53 (2.34–5.32)	7 (4.60–10.67)	3.82 (2.83–5.15)	
**Age- and sex-adjusted HR (95% CI) for all-cause mortality**	1	1.05 (0.75–1.47)	1.57 (1.04–2.39)	2.20 (1.43–4.00)	1.78 (1.31–2.42)	
Multivariate^*^ **for all-cause mortality**	1	0.99 (0.71–1.39)	1.51 (1.00–2.28)	2.02 (1.29–3.16)	1.71 (1.25–2.34)	

*Previously adjusted by age, sex, previous cardiovascular disease, smoking habits, history of high blood pressure, low-density lipoprotein cholesterol, estimation of glomerular filtration rate and body mass index.

Abbreviations: T2D, type 2 diabetes; CI, confidence interval; HR, hazard ratio.

**Fig 2:**
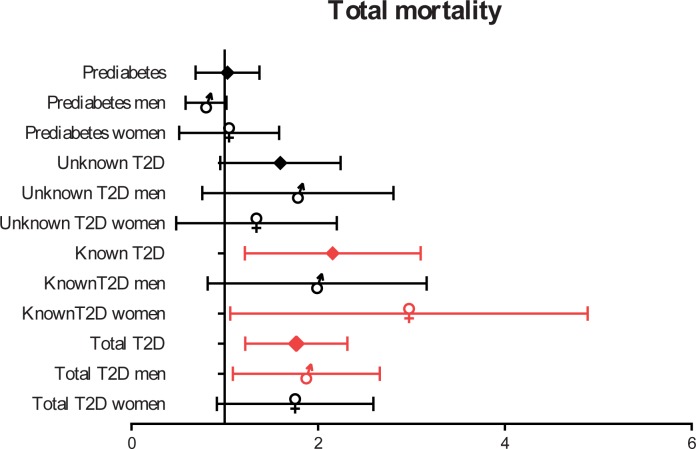
HR (95% CI) for all-cause mortality depending on gender and presence of known or undiagnosed T2D. Model adjusted for gender, age, body mass index, history of previous cardiovascular disease, history of high blood pressure, smoking, low density lipoprotein cholesterol and estimated glomerular filtration rate.

The age- and sex-adjusted mortality risks were almost equal in the NGT and prediabetes groups and higher in subjects with previously undiagnosed T2D; however, the risks were not as high as for known T2D at baseline (Table 2). After multivariable adjustment, people with known T2D had a mortality rate (MR) more than two-fold greater than people in the NGT group (2.02 [95% CI 1.29–3.16]); whereas, for people with undiagnosed T2D, the MR was 1.51-times higher (95% CI 1.00–2.28) (Fig 2).

The risk of death in those with T2D, both diagnosed and undiagnosed, versus those without dysglycemia was 1.71 (95% CI 1.25–2.34) in the multivariate model and 1.78 (95% CI 1.31–2.42) in the model adjusted for age and sex.

### Cause of death, age, and sex differences

Individuals with T2D presented with higher risks of cardiovascular and cancer death in the 18-year follow-up. In adjusted models, cancer mortality was slightly increased in subjects with known and undiagnosed T2D, whereas cardiovascular mortality was highly increased in both groups (Table 3).

**Table 3:** Hazard ratios for all-cause, cardiovascular, and cancer mortality based on dysglycemia categories.

	All-cause mortality			CV mortality			Cancer mortality		
	HR	95% CI	P	HR	95% CI	P	HR	95% CI	P
**Dysglycemia Categories**									
**Euglycemia**	1			1			1		
**Prediabetes** Men Women	0.99 0.89 0.96	0.71–1.39 0.55–1.41 0.56–1.62	0.95 0.61 0.87	1.07 1.35 1.04	0.59–1.94 0.58–3.15 0.44–2.50	0.83 0.49 0.92	0.78 0.85 0.64	0.43–1.40 0.41–1.75 0.23–1.81	0.40 0.66 0.40
**Known T2D** Men Women	**2.02** 1.76 **2.56**	1.29–3.16 0.95–3.26 1.30–5.06	0.002 0.130 0.007	**2.79** 1.66 **6.72**	1.35–5.75 0.46–5.95 2.50–18.07	0.006 0.44 0.001	1.77 2.30 1.70	0.78–4.03 0.85–6.22 0.36–8	0.17 0.10 0.50
**Unknown T2D** Men Women	1.51 1.59 1.15	1.00–2.28 0.87–2.89 0.60–2.28	0.050 0.130 0.580	1.44 2.06 1.93	0.67–3.08 0.60–7.04 0.66–5.63	0.35 0.25 0.23	1.33 1.68 0.68	0.65–2.74 0.69–4.08 0.17–1.81	0.43 0.25 0.58
**Total T2D** Men Women	**1.71** **1.76** 1.62	1.25–2.34 1.15–2.71 0.99–2.65	0.001 0.010 0.055	**1.82** 1.57 **3.31**	1.09–3.03 0.68–3.65 1.59–6.89	0.027 0.29 0.001	1.67 **2.04** 1.09	0.96–2.90 1.03–4.04 0.38–3.12	0.07 0.04 0.87

Abbreviations: CV, cardiovascular; HR, hazard ratio; CI, confidence interval; T2D, type 2 diabetes. The statistically significant results are marked in bold.

Individuals who died of cancer were younger than those who died from other causes. Previous cardiovascular disease, a higher level of HbA1c, and a history of hypertension predicted deaths from cardiovascular events. Furthermore, smoking was more common in people who died of cancer (Table 4).

**Table 4:** Differences in metabolic and biochemical data according to causes of mortality.

	CV mortality (74)	Cancer mortality (72)	Others (58)	P-value
**Age at death, years**	**77.64±9.78**	**73.75±10.95**	**77.91±11.65**	**0.041**
**eGFR (ml/min)**	68.40±18.13	69.01±16.28	69.32±16.49	0.453
**LDL cholesterol (mg/mL)**	160.58±45.75	149.15±42.86	156.47±42.13	0.291
**BMI (kg/m**^**2**^**)**	28.87±5.33	28.11±4.14	28.65±4.41	0.602
**HbA1c (%)**	**6.03±1.56**	**5.43±0.83**	**5.53±1.14**	**0.007**
**Previous CVD (%)**	**18 (24.3)**	**8 (11.1)**	**4 (6.9)**	**0.011**
**Men (%)**	35 (47.3)	47 (65.3)	29 (50)	0.07
**Smokers (%)**	11 (14.9)	19 (26.4)	8 (13.8)	0.08
**Previous HBP (%)**	**39 (52.7)**	**24 (33.3)**	**17 (29.3)**	**0.01**
**No sport (%)**	70 (94.6)	64 (88.9)	52 (89.6)	0.43
**SBP (mmHg)**	151.35±20.65	148.74±22.16	144.36±25.24	0.21
**DBP (mmHg)**	88.62±13.27	89.64±14.87	86.60±13.86	0.46
**Total cholesterol (mg/dL)**	240.59±50.04	227.42±47.03	241.31±45.85	0.16
**HDL cholesterol (mg/dL)**	53.02±13.20	53.87±14.23	58.30±14.40	0.08
**Triglycerides (mg/dL)**	141.04±77.22	123.39±56.70	132.69±70.25	0.31
**Estimated fat index (%)**	37.40±8.73	34.21±8.50	36.80±8.55	0.06

CV, cardiovascular; eGFR, estimated glomerular filtration rate,; LDL, low-density lipoprotein; BMI, body mass index; HbA1c, glycohemoglobin; CVD, cardiovascular disease; HBP, high blood pressure; SBP, systolic blood pressure; DBP, diastolic blood pressure; HDL, high-density lipoprotein. The statistically significant results are marked in bold.

Moreover, persons with a history of cardiovascular disease had a higher risk of all-cause (and especially cardiovascular) mortality, with statistical significance. HRs for persons with previously known T2D compared to those in the NGT group were higher in women than in men (2.56 vs. 1.76). Furthermore, men with newly diagnosed T2D had a higher risk of cardiovascular death than men with previously diagnosed T2D.

**Fig 3:**
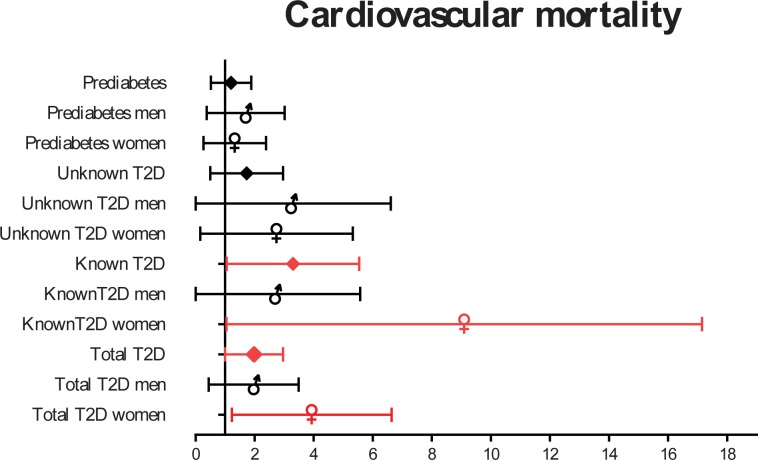
HR (95% CI) for cardiovascular mortality depending on gender and presence of known or undiagnosed T2D.

The HR for cardiovascular mortality in women with known T2D was 6.72 (95% CI 2.50–18.07) vs. 1.66 (95% CI 0.46–5.95) in men with the same condition (Fig 3).

**Fig 4:**
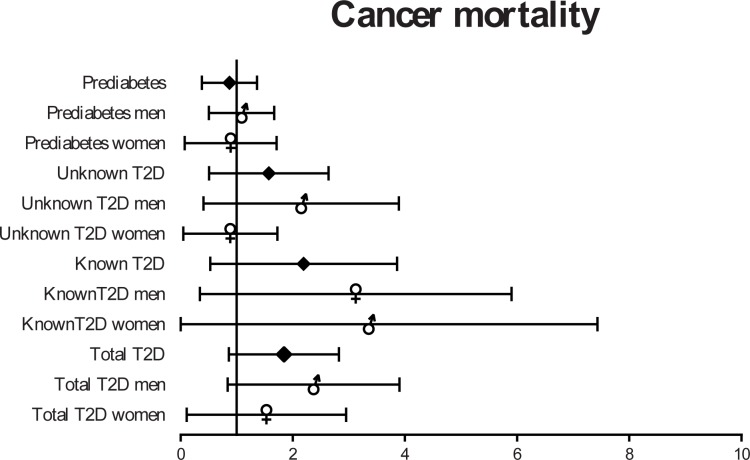
HR (95% CI) for Cancer mortality depending on gender and presence of known or undiagnosed T2D.

Cancer mortality was also increased in people with known T2D, especially in men; however, it was not statistically different (Fig 4).

People younger than 45 years with T2D (both diagnosed and unknown) had a strongly increased mortality risk compared to the NGT group of the same age. This risk decreased with increasing age (Table 5).

**Table 5:** Hazard ratios (95% confidence interval) for all-cause mortality in persons with type 2 diabetes depending on age group.

	T2D			Known T2D			Unknown T2D			N
	Adjusted HR for all-cause mortality (CI 95%)	N^†^	n	Adjusted HR for all-cause mortality (CI 95%)	N^†^	n	Adjusted HR for all-cause mortality (CI 95%)	N^†^	n	
**30–45 years**	15.91 (2.4–105.58)	2	10	38.02 (2.79–518.33)	1	1	8.19 (0.6–111.85)	1	9	338
**45–60 years**	1.48 (0.5–4.36)	7	30	3.67 (0.76–17.68)	3	8	0.81 (0.22–2.97)	4	22	323
**≥60 years**	1.64 (1.16–2.31)	53	91	1.94 (1.19–3.18)	26	39	1.4 (0.86–2.29)	27	52	373

N^†^: Number of dead participants in each age group.

Abbreviations: T2D, type 2 diabetes; HR, hazard ratio; CI, confidence interval.

## Discussion

In this Spanish population-based sample, regionally representative with up to 18-year follow-up, mortality risk was more than two-times higher (2.02) among adults with known T2D than among adults in the NGT group. Risk of death associated with T2D was especially increased in younger age groups. Moreover, mortality risk was notably higher in women with T2D than in men (both with known and undiagnosed T2D) because of an increase in cardiovascular mortality.

Although previous studies reported different data, comparability is limited because of differences in numbers, age, and sex structures. In 2017, Röckl et al.[14] published a similar study with 6550 participants (575 with T2D) that showed that mortality was almost twice as high in the T2D group than in the NGT group; however, mortality tended to be higher in men than in women in the case of undiagnosed T2D. Tancredi et al. [15] followed 435,369 Swedish persons with T2D aged ≥18 years from 1998–2011 and compared their mortality to matched controls. The HR for mortality was 1.77 (95% CI 1.03–3.04) in the T2D group. The KORA S4 study compared a population of 1466 German adults aged 55–74 years and reported higher mortality rates in people with known T2D than for those in the NGT group: 2.6 (95% CI 1.7–3.8) [16]. The ERFORT study (1125 German men aged 40–59 years) compared men with known T2D and normoglycemia. Mortality risks ranged from 1.86 (95% CI 1.22–2.84) to 2.22 (95% CI 1.36–3.63) depending on the duration of follow-up [17].

Lind et al. [18] used healthcare databases in Canada and the United Kingdom (UK) to calculate annual MRs for adults aged ≥20 years with and without T2D from 1996–2009. HRs for mortality were higher for those with T2D: 2.68 (95% CI 1.51–4.74) in Canada and 2.41 (95% CI 1.28–4.53) in the UK. As reported previously in the National Health and Nutrition Examination Survey (NHANES) II study [19,20], the HR for cardiovascular mortality was smaller in undiagnosed diabetes than in diagnosed diabetes (1.54 [95% CI 0.85–2.79] and 2.62 [95% CI 1.81–3.78], respectively). The results of our analysis of cardiovascular death risk showed a significant association with known T2D but not with newly diagnosed T2D. This finding could be due to the small sample size and the limited duration of diabetes mellitus.

As stated earlier, comparability with other studies is problematic because our sample size was smaller; however, HRs for mortality in T2D groups were similar to those obtained in the UK. Our analyses showed higher HRs for mortality in women with T2D than in men, specifically due to cardiovascular disease as reported previously [21–24].

Furthermore, our study showed a similar increase in cancer mortality in both the previously known and unknown T2D groups, and this finding was not significantly different from that of the NHANES II study. FRESCO [25], a Spanish study of 55,292 individuals, showed that diabetes was associated with premature death from cardiovascular disease and cancer, in keeping with our results.

According to our data, subjects with newly diagnosed T2D have a lower mortality risk than those with known T2D. This may be due in part to the shorter time of evolution and the improved profile of metabolic and lifestyle risk factors at baseline. Subjects with undiagnosed T2D had lower systolic and diastolic blood pressure and better eGFR than those with previously known T2D, but higher LDL-cholesterol. A possible explanation for the last finding is the greater use of lipid-lowering drugs in persons with diagnosed T2D.

Mortality risk in this cohort was studied in 2005 by Valdés et al. [9] after 6 years of follow-up. According to their results at that time, both individuals with diagnosed diabetes and those with undiagnosed diabetes had a risk of mortality around 2.5-3-times greater than individuals with normoglycemia. Compared with our results, the increase in mortality in persons with T2D was maintained. The importance of the time of evolution of diabetes became even more evident in our study, as people who were diagnosed in 1998 had lower MRs than those diagnosed before 1998.

In addition, according to a previous study [26], prediabetes (IGT or IFG according to IFG-ADA or IFG-WHO criteria) is associated with an increased risk of composite cardiovascular disease (relative risk 1.13, 1.26, and 1.30 for IFG-ADA, IFG-WHO, and IGT, respectively) and all-cause mortality (1.13, 1.13, and 1.32, respectively). However, another previous study [27] found that prediabetes (those who did not indicate diagnosed diabetes and had HbA1C 5.7%-<6.5% [39-<48 mmol/mol]) was not associated with increased mortality, and raised HbA1c did not appear to have a statistically significant impact upon cancer mortality. Our data showed similar results; the prediabetes group did not have an increased MR compared to the NGT group using ADA 2018 criteria. This absence of association may be because we sent letters to all individuals with their results, encouraging them to incorporate lifestyle changes if they had any alterations in glucose metabolism.

Several limitations apply to this study. The main limitation is the small sample size, with a limited number of events available for analysis. Thus, it was difficult to demonstrate statistical differences. Nevertheless, for cases in which we able to show statistical significance, the effect can be considered large. However, this study has several strengths, including that it was population-based, and the mortality follow-up was relatively long. Moreover, cardiovascular and lifestyle risk factors were included to calculate the multivariate adjustment of the regression models.

## Conclusion

Individuals with diabetes (especially those with diagnosed diabetes) had a significantly higher mortality risk than those with NGT after 18 years of follow-up even after adjusting for multiple confounders. Excess mortality was especially noticeable in younger women, predominantly due to cardiovascular disease.

Our results highlight the need for an early diagnosis and intensive multifactorial treatment considering the present diabetes pandemic.

## Abbreviations

T2D: Type 2 diabetes
HR: Hazard ratio
MR: Mortality Rate
HbA1c: Glycohemoglobin
BMI: Body mass index
BP: Blood pressure
OGTT: Oral glucose tolerance test
HDL: High-density cholesterol
LDL: Low-density cholesterol
HPLC: High power liquid chromatography
ADA: American Association of Diabetes
IGT: Impaired glucose tolerance
IFG: Impaired fasting glucose
NGT: Normal glucose tolerance
BF: Body fat
RR: Relative risk
