# Correction: Mortality risk in adults according to categories of impaired glucose metabolism after 18 years of follow-up in the North of Spain: The Asturias Study

**DOI:** 10.1371/journal.pone.0216629

**Published:** 2019-05-09

**Authors:** Jessica Ares, Sergio Valdés, Patricia Botas, Cecilia Sánchez-Ragnarsson, Sandra Rodríguez-Rodero, Paula Morales-Sánchez, Edelmiro Menéndez-Torre, Elías Delgado

Fig 2, “HR (95% CI) for all-cause mortality depending on gender and presence of known or undiagnosed T2D,” is incorrect. Please see the complete, correct Fig 2 here.

**Fig 2:**
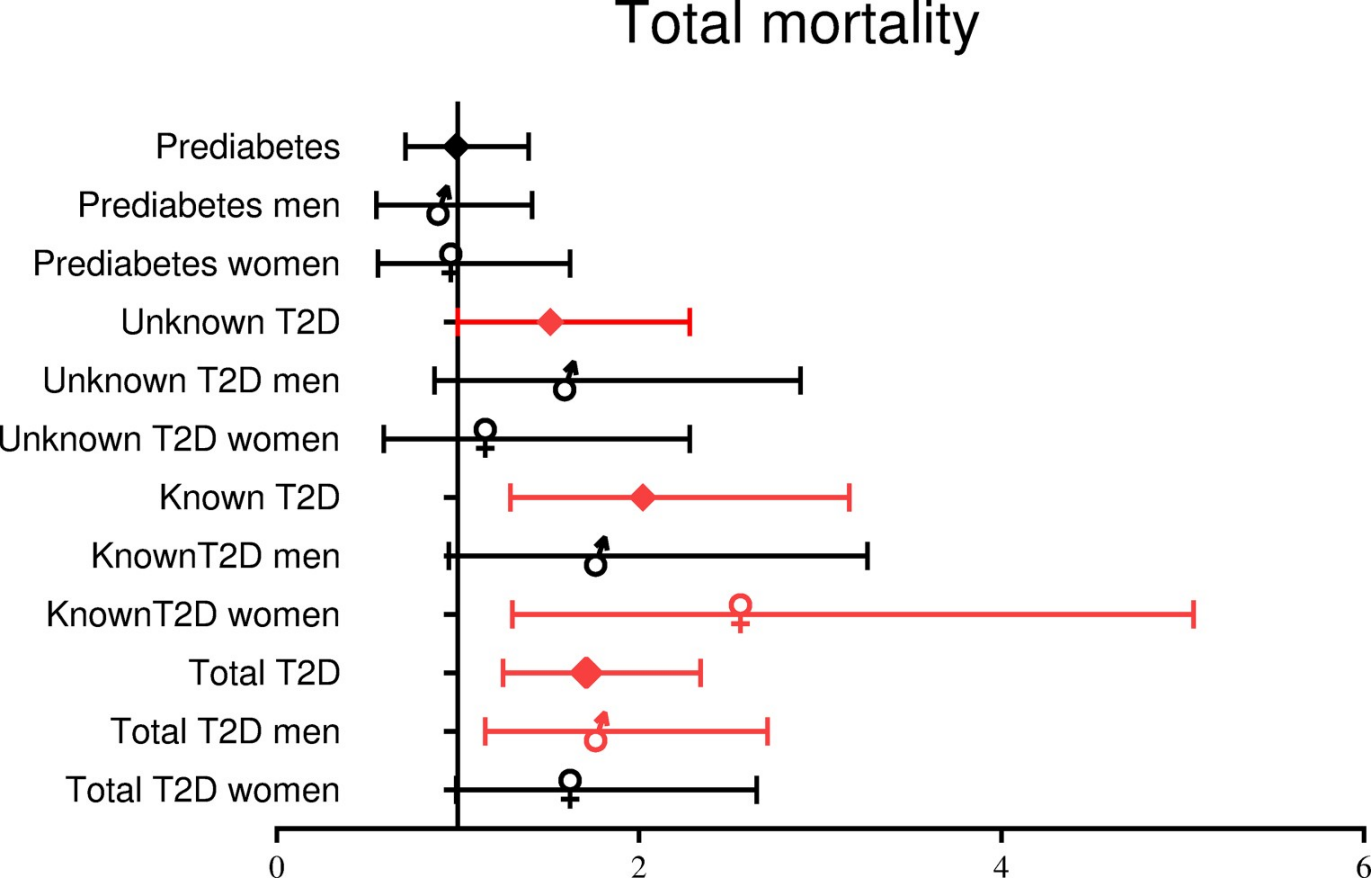
HR (95% CI) for all-cause mortality depending on gender and presence of known or undiagnosed T2D. Model adjusted for gender, age, body mass index, history of previous cardiovascular disease, history of high blood pressure, smoking, low density lipoprotein cholesterol and estimated glomerular filtration rate.

Fig 3, “HR (95% CI) for cardiovascular mortality depending on gender and presence of known or undiagnosed T2D,” is incorrect. Please see the complete, correct Fig 3 here.

**Fig 3:**
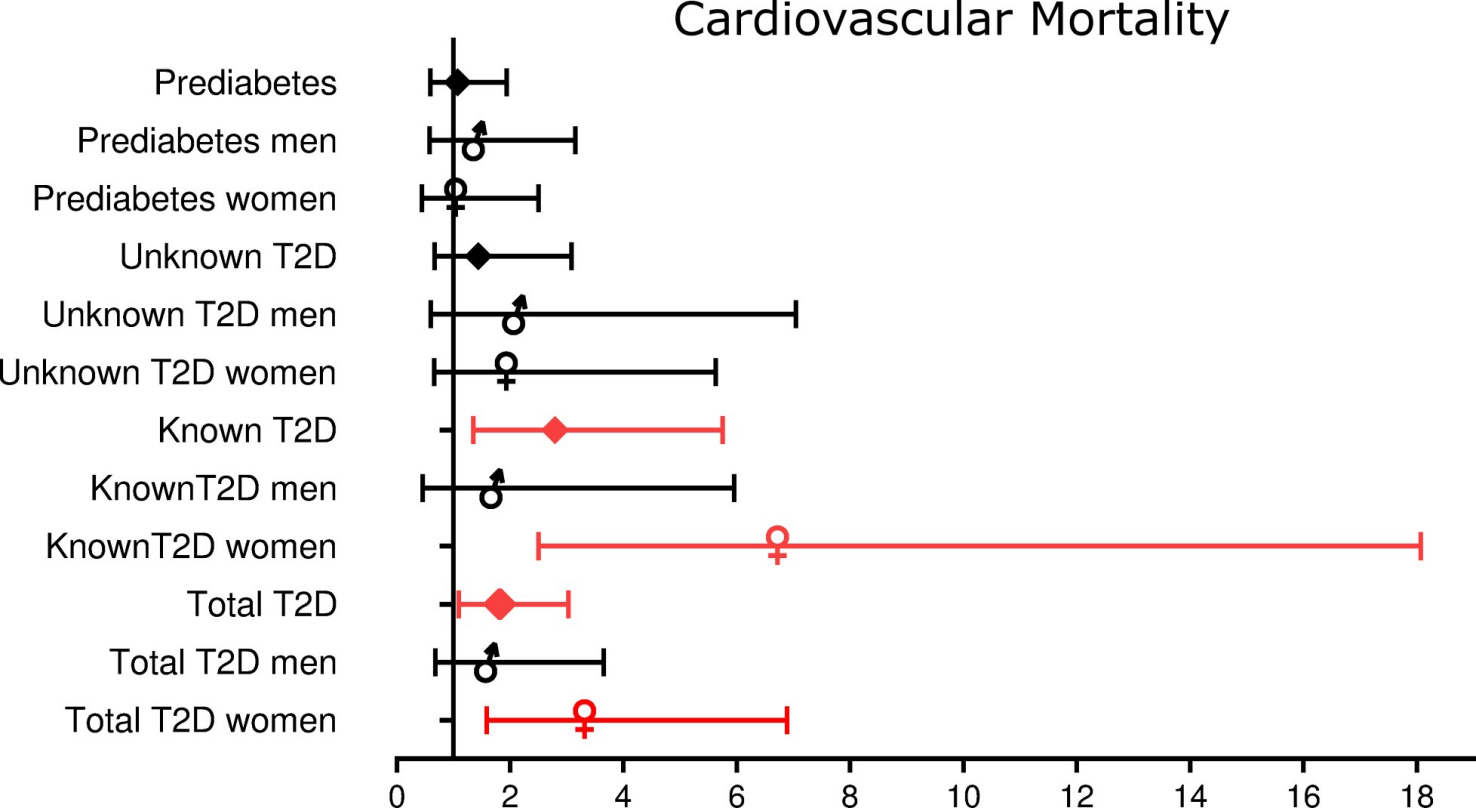
HR (95% CI) for cardiovascular mortality depending on gender and presence of known or undiagnosed T2D.

Fig 4, “HR (95% CI) for Cancer mortality depending on gender and presence of known or undiagnosed T2D,” is incorrect. Please see the complete, correct Fig 4 here.

**Fig 4:**
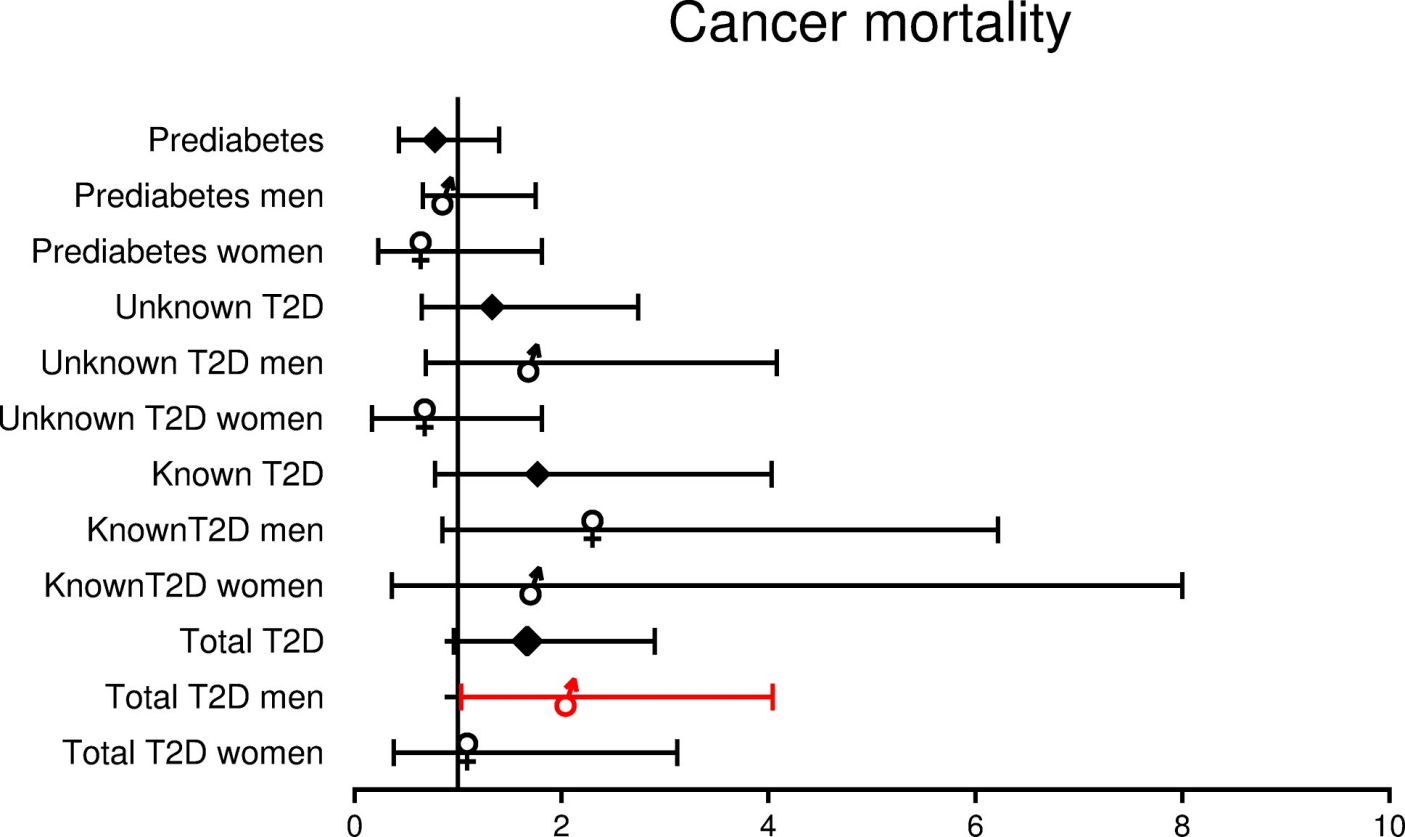
HR (95% CI) for Cancer mortality depending on gender and presence of known or undiagnosed T2D.
